# Targeted capture-based NGS is superior to multiplex PCR-based NGS for hereditary *BRCA1* and *BRCA2* gene analysis in FFPE tumor samples

**DOI:** 10.1186/s12885-019-5584-6

**Published:** 2019-04-27

**Authors:** Falk Zakrzewski, Laura Gieldon, Andreas Rump, Michael Seifert, Konrad Grützmann, Alexander Krüger, Sina Loos, Silke Zeugner, Karl Hackmann, Joseph Porrmann, Johannes Wagner, Karin Kast, Pauline Wimberger, Gustavo Baretton, Evelin Schröck, Daniela Aust, Barbara Klink

**Affiliations:** 1 Core Unit for Molecular Tumor Diagnostics (CMTD), National Center for Tumor Diseases (NCT), Schubertstraße 15, 01307 Dresden, Germany; 2 Institute for Clinical Genetics, Medical Faculty Carl Gustav Carus, Technische Universität Dresden, Dresden, Germany; 3 Institute for Medical Informatics and Biometry (IMB), Medical Faculty Carl Gustav Carus, Technische Universität Dresden, Dresden, Germany; 4 National Center for Tumor Diseases (NCT), Dresden, Germany; 5 Institute of Pathology, University Hospital Carl Gustav Carus Dresden, Dresden, Germany; 6 German Cancer Consortium (DKTK), Dresden, Germany; 7 Department of Gynecology and Obstetrics, University Hospital Carl Gustav Carus Dresden, TU Dresden, Dresden, Germany; 8 Tumor- and Normal Tissue Bank of the University Cancer Center (UCC), University Hospital Carl Gustav Carus Dresden, Technische Universität Dresden, National Center for Tumor Diseases (NCT) Dresden, Dresden, Germany

**Keywords:** HBOC, Genetic testing, Pathogenic germline mutations, NGS, *BRCA1*, *BRCA2*, FFPE tissue, Targeted capture-based NGS, CNV detection

## Abstract

**Background:**

With the introduction of Olaparib treatment for BRCA-deficient recurrent ovarian cancer, testing for somatic and/or germline mutations in *BRCA1/2* genes in tumor tissues became essential for treatment decisions. In most cases only formalin-fixed paraffin-embedded (FFPE) samples, containing fragmented and chemically modified DNA of minor quality, are available. Thus, multiplex PCR-based sequencing is most commonly applied in routine molecular testing, which is predominantly focused on the identification of known hot spot mutations in oncogenes.

**Methods:**

We compared the overall performance of an adjusted targeted capture-based enrichment protocol and a multiplex PCR-based approach for calling of pathogenic SNVs and InDels using DNA extracted from 13 FFPE tissue samples. We further applied both strategies to seven blood samples and five matched FFPE tumor tissues of patients with known germline exon-spanning deletions and gene-wide duplications in *BRCA1/2* to evaluate CNV detection based solely on panel NGS data. Finally, we analyzed DNA from FFPE tissues of 11 index patients from families suspected of having hereditary breast and ovarian cancer, of whom no blood samples were available for testing, in order to identify underlying pathogenic germline *BRCA1/2* mutations.

**Results:**

The multiplex PCR-based protocol produced inhomogeneous coverage among targets of each sample and between samples as well as sporadic amplicon drop out, leading to insufficiently or non-covered nucleotides, which subsequently hindered variant detection. This protocol further led to detection of PCR-artifacts that could easily have been misinterpreted as pathogenic mutations. No such limitations were observed by application of an adjusted targeted capture-based protocol, which allowed for CNV calling with 86% sensitivity and 100% specificity. All pathogenic CNVs were confirmed in the five matched FFPE tumor samples from patients carrying known pathogenic germline mutations and we additionally identified somatic loss of the second allele in *BRCA1/2*. Furthermore we detected pathogenic *BRCA1/2* variants in four the eleven FFPE samples from patients of whom no blood was available for analysis.

**Conclusions:**

We demonstrate that an adjusted targeted capture-based enrichment protocol is superior to commonly applied multiplex PCR-based protocols for reliable *BRCA1/2* variant detection, including CNV-detection, using FFPE tumor samples.

**Electronic supplementary material:**

The online version of this article (10.1186/s12885-019-5584-6) contains supplementary material, which is available to authorized users.

## Background

Pathogenic germline mutations in *BRCA*1 and *BRCA*2 can be identified as the underlying cause in more than 10% of hereditary breast and ovarian cancer (HBOC) cases. Female mutation carriers have an estimated risk of 50–80% for developing breast cancer and 30–50% [1–3] for ovarian-cancer. Consequently, close clinical surveillance from an early age as well as prophylactic operations are recommended for female mutation carriers. BRCA1 and BRCA2 proteins are linked within a network of protein interactions that responds to DNA damage [4]. Disruption of key elements of DNA-repair, such as BRCA1 and BRCA2, leads to genomic instability and sensitivity to DNA damage, such as double-strand breaks [5, 6]. Tumors with impaired DNA damage response (DDR) respond to treatment with PARP-inhibitors, such as Olaparib, an oral inhibitor of poly (ADP-ribose) polymerase (PARP) proteins, which plays a key role in DNA repair and genomic stability. Olaparib is now routinely applied as maintenance treatment for patients with platinum-sensitive recurrent, *BRCA1/2*-mutated ovarian cancer. Additionally, the drug is currently being tested in several clinical trials in different tumor entities with underlying mutations in *BRCA1/2* and other genes involved in DDR. Therefore reliable diagnostic tests for the detection of *BRCA1/2* mutations and variants in other genes involved in DDR in tumor tissues are crucial for treatment decision making [1, 7, 8].

Families with a high risk for HBOC are commonly tested for *BRCA*1 and *BRCA*2 germline variants by next-generation sequencing (NGS) complemented by array CGH or MLPA for copy number variation (CNV) detection using blood samples, saliva samples, or buccal smear in order to obtain high-quality DNA [1, 7–9]. Even in HBOC-families, however, tumor analysis can be necessary, e.g. if the index patient is deceased or if mosaicism is suspected. In addition, testing of tumor tissues aids interpretation of variants of unknown significance (VUS) by identification of a possible second hit, which could be either a loss-of-heterozygosity (LOH) or a pathogenic somatic variant disrupting the second allele. Since paraffin-embedding is routinely applied for tissue preservation, formalin-fixed, paraffin-embedded (FFPE) tissues are often stored for several years and are available for genetic testing. Reliable analysis pipelines applicable to FFPE samples therefore are of great diagnostic importance.

Identification of pathogenic *BRCA1/2* variants is challenging due to the large size of these genes. The coding regions of *BRCA1* and *BRCA2* span up to ~ 7.2 kb and ~ 11.4 kb, respectively [10–14]. Since the common mechanism of pathogenicity of mutations in DNA damage response genes, such as *BRCA1/2*, is a loss of function, all exons (and potential splice sites) need to be analyzed, resulting in a large set of target regions. Moreover, detection of SNVs or small InDels by panel sequencing is mostly insufficient and diagnostic algorithms therefore need to be complemented by CNV detection.

So far, more than 6200 unique *BRCA* variants are known, including a total of 1826 pathogenic or likely pathogenic variants and 2828 VUS [15]. In addition to point mutations, a range of CNVs causing HBOC have been identified. We previously described six HBOC families in whom we detected different *BRCA1/2* CNVs using custom array CGH [8]. However, array CGH or MLPA is not applicable to highly fragmented FFPE-derived DNA, leaving CNV detection based on NGS data as the only possible approach.

Sequencing and subsequent analysis of DNA from FFPE material is complicated by the high fragmentation grade of formalin-treated DNA and by modification of nucleic acids by protein-nucleic acid and protein-protein cross linking [16, 17]. Therefore, a variety of different protocols have been developed, mostly based on multiplex-PCR, for mutation testing in selected regions of the *BRCA1/2* genes. Here, we compare the commonly used multiplex PCR-based NGS approach (BRCA DNA Repair Panel - TruSeq Custom Amplicon LowInput panel (Illumina Inc.)) to a targeted capture-based approach (an adjusted protocol corresponding to the TruSight Cancer panel pipeline (Illumina Inc.)). We demonstrate that the targeted capture-based NGS strategy is superior with respect to overall performance, reliable pathogenic variant detection, coverage homogeneity of each sample and among samples, the absence of allele drop outs and non-covered nucleotides, as well as false positive variants/artifacts. Most notably, reliable identification of CNVs in *BRCA1/2* was possible using DNA derived from FFPE tissues by application of this novel approach.

## Methods

### Acquisition of patient material

An overview of all patients included in this study, the particular samples investigated for *BRCA1/2* variants as well as additional information (tumor entity and TNM staging where provided) is given in Additional file 4: Table S3 Blood-samples were acquired within a diagnostic setting from all available index patients. FFPE tissue was provided by the Institute for Pathology at the University Hospital Dresden. Patients were counselled prior to genetic analyses and informed consent was obtained from all patients in accordance with the German Gene Diagnostics Act (GenDG). Tumor tissue was micro-dissected and tumor cell content was determined by a pathologist.

### Preparation of DNA from blood and from FFPE samples

DNA from blood samples was prepared using the QIAmp DNA Blood Midi kit (Qiagen, Inc.) and DNA from micro-dissected FFPE material was prepared using the QIAamp DNA Micro Kit (Qiagen, Inc.) according to the manufacturer’s instructions. DNA concentrations were determined via Qubit (Thermo Fisher, Inc.) measurement and the degree of fragmentation was determined via qPCR (KAPA SYBR® FAST qPCR). For the targeted capture-based enrichment protocol 50–200 ng of DNA were randomly sheared to fragment sizes of ca. 170–240 bp using the Covaris ultrasonciator (Covaris, Inc.) and fragment size was verified using a Fragment Analyzer (Advanced Analytical, Inc.).

### Library generation and sequencing

Library construction for targeted capture-based NGS was modified and performed using the TruSeq Nano DNA Library Prep Kit (Illumina, Inc.) instead of routinely applied Transposome-based library generation. Since DNA obtained from FFPE samples often is highly fragmented, the TruSeq Nano DNA Library Prep Kit works more efficiently for library generation compared to the Transposome-based protocol. After the first PCR step the TruSight Cancer (Illumina, Inc.) protocol was applied (referred to as targeted capture-based NGS/enrichment in this study), which targets the coding sequences of 94 genes associated with a cancer predisposition [8]. For multiplex PCR-based NGS the *BRCA* DNA Repair Panel - TruSeq Custom Amplicon LowInput panel (Illumina, Inc.), referred to as multiplex PCR-based NGS in this study, was used. Library size was controlled by Fragment Analyzer (Advanced Analytical, Inc) measurements. Both targeted capture-based NGS and multiplex PCR-based NGS were conducted on a MiSeq sequencing platform with 2 × 150 bp paired-end runs using Mid Output V2 (Illumina, Inc.) chemistry.

### Mapping, variant detection and visualization

Mapping against the human reference genome sequence hg19-Ensembl (GRCh37/hg19) was performed using the CLC Biomedical Genomics Workbench v.3.5 [18] (CLC BMW). Major parameters were set as follows: match score 1; mismatch cost 2; affine gap cost (insertion/deletion open cost 6; insertion/deletion extend cost 1); length fraction 0.5; similarity fraction 0.8. Reads that mapped equally at multiple sites (e.g. repetitive regions) were discarded. Variants were detected by the low frequency variant detection tool of the CLC BMW set to default parameters. Calling was restricted to target regions provided by Illumina (cf. BED file) on the panel specific website. Potential splice site variants were analyzed +/− 2 nucleotides from the corresponding exon. Variants were annotated with ClinVar, COSMIC v.78 and ExAC v0.3 variant information. Variants were manually evaluated in accordance with ACMG criteria for determination of pathogenicity [19]. For targeted capture-based NGS variants were regarded as sufficiently covered if at least 30 reads mapped at the corresponding position. For multiplex PCR-based NGS coverage of each nucleotide with at least 500 reads was considered sufficient. Plots displaying normalized coverage along all concatenated targets were generated using R custom scripts [20]. Normalization against median coverage was performed at single nucleotide level and for each sample individually.

### Detection of CNVs and visualization

CNV detection based on targeted capture-based NGS data was performed using the R-package panelcn.MOPS [21] set to default parameters. Panelcn.MOPS is based on the genome-wide and whole exome-wide CNV detection tool cn.MOPS [22]. cn.MOPS builds a local model that captures the read characteristics of each region of interest, avoiding a bias induced by the targeting procedure [21]. We modified the package output to be reported as log2 values rather than custom values provided by the standard settings of the package. Thirty five blood samples that were sequenced using the same targeted panel (TSC94) and that did not show any CNVs in complementary array CGH analysis were used as control data set. The complete target regions of all 94 genes (including *BCRA1/2*) of these 35 samples were used for normalization and as controls for the panelcn.MOPS pipeline. Probes spanning an individual region with a size of more than 300 nucleotides were subdivided into smaller targets with at least 70–100 nucleotides and a maximum of 200 nucleotides to increase the resolution of CNV detection. Results (including log2 values) were visualized using the plot function of panelcn.MOPS. In addition, a csv-file was created for each sample, displaying statistical parameters and copy number changes (CN; CN0 = loss; CN1 = one copy; CN2 = two copies; CNx = x copies) for each target (Additional file 5: Excel spreadsheet 1).

## Results

### Quality improvement of *BRCA1/2* panel NGS of FFPE-sample derived DNA by adjusted targeted capture-based enrichment

We routinely apply the TruSight Cancer Panel (Illumina, Inc.) for germline variant analysis in HBOC families. Library preparation includes a so called tagmentation step in which high-quality, blood-derived DNA is sheared by a transposase and a capture step enriching 94 cancer-associated genes including *BRCA1/2*. In order to apply the 94 gene panel to low quality FFPE samples, we implemented an adapted strategy that relies on random fragmentation of DNA using the Covaris ultrasonicator (Covaris, Inc.) followed by application of the TruSeq DNA Nano kit (Illumina, Inc.) prior to enrichment via the TruSight Cancer Panel. The tagmentation step, which is usually included in the TruSighCancer preparation protocol, was skipped. To compare the performance of the modified targeted capture-based protocol to a multiplex PCR-based strategy (*BRCA* DNA Repair Panel / TruSeq Custom Amplicon LowInput panel kit (Illumina, Inc.)), we first investigated the overall quality of both NGS approaches using DNA obtained from five high-quality blood control samples from healthy individuals. This was followed by the investigation of low quality DNA from 13 FFPE ovarian tumor samples from 12 patients with previously known germline mutations in *BRCA1/2* and one patient in whom no germline mutation had been detected despite HBOC family history (Tab. 1).

**Table 1:** Comparison of the targeted capture-based to the multiplex PCR-based NGS strategy applied to DNA originating from FFPE tissue samples from 13 ovarian cancers

ID	Material	Sample group	Known pathogenic variant in blood	Multiplex PCR-based NGS		Targeted capture-based NGS	
				Pathogenic variant detected?^a^	False positive path. Variants	Pathogenic variant detected?^a^	False positive path. Variants
P01	FFPE tissue	Routine diagnostic	BRCA2:p.Ala1327fs	yes	3	yes	0
P02			BRCA2:p.Asn1747fs	yes	3	yes	0
P03			BRCA1:p.Gln1756fs	yes	3	yes	0
P04			BRCA1:p.Leu786fs	yes	3	yes	0
P05			BRCA1:p.Cys61Gly	(no)	3	yes	0
P06			BRCA2:p.Val1283fs	yes	3	yes	0
P07			BRCA2:p.Asn433fs	yes	3	yes	0
P08			BRCA2:p.Asn1747fs	yes	3	yes	0
P09			–	no^b^	3	no^b^	0
P10			BRCA1:NM_007294.3:c.4675 + 1G > A	(no)	2	yes	0
P11			BRCA1:p.Glu23fs	yes	3	yes	0
P12			BRCA2:p.Asn2135fs	yes	3	yes	0
P13			BRCA1:NM_007294.3:c.4675 + 1G > A	yes	3	yes	0

^a^See Additional file 3: Table S2 for detailed information on type of pathogenic variant

^b^no pathogenic germline BRCA1/2 variant detected in blood from the same patient

Multiplex-based NGS (Additional file 2: Table S1) of blood control samples resulted in occurrence of low-covered and non-covered nucleotides (< 500 was considered low-covered in accordance with generally applied minimum read counts for nucleotides in multiplex PCR-based approaches [23, 24]). Since array CGH did not detect any CNVs in these samples (data not shown), these low- or non-covered nucleotides were interpreted as artifacts occurring as a result of amplicon drop out. In contrast, all nucleotides were covered with at least the minimum amount of reads necessary for reliable variant detection with the modified targeted capture-based approach (> 30 was considered low-covered in accordance with the generally applied minimum read count for nucleotides in targeted capture-based approaches for germline samples [19, 25]). We used criteria for the multiplex PCR-based technique routinely applied in daily diagnostic of somatic cancer tissue. Therefore, higher read depths used in the multiplex PCR-based protocol compared to the targeted enrichment-based protocol (500 vs. 30) is due to necessity for confident identification of somatic variants in tumor specimens because of cancer tissue heterogeneity [26].

**Fig. 1:**
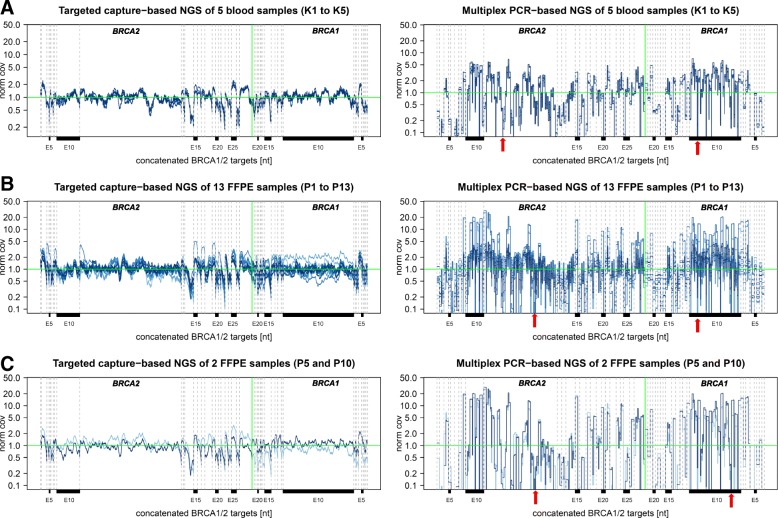
Comparison of normalized coverage of targeted capture-based NGS and multiplex PCR-based NGS in control blood and diagnostic FFPE tumor samples. **a** Normalized coverage (y-axis) of targeted capture-based and multiplex PCR-based NGS at single-base resolution along all concatenated *BRCA1/2* targets (x-axis) of five blood control samples (Additional file 3: Table S2, K1 to K5). The five blood samples are color-coded. The vertical green line indicates the end of *BRCA*1 targets and the start of the *BRCA*2 targets (target number is increasing from left to right which corresponds to five to three prime orientation of the gene). The horizontal green line displays normalized coverage of 1.0. All target exons are separated via gray dotted vertical lines. Selected target exons are marked. Exemplary, two randomly chosen amplicon dropouts are marked by a red arrow. **b** Normalized coverage (y-axis) of targeted capture-based and multiplex PCR-based NGS of FFPE samples from patient 1 to 15 (Additional file 3: Table S2, P01 to P15) at single-base resolution along all concatenated *BRCA1/2* targets (x-axis) Samples P1 to P13 are color encoded in blue. Exemplary, randomly chosen capture target dropouts and amplicon dropouts are marked by a red arrow. The vertical green line indicates the end of the *BRCA2* targets (target number is increasing from left to right which corresponds to five to three prime orientation of the gene) and the start of the *BRCA1* targets (decreasing from left to right). The horizontal green line displays normalized coverage of 1.0. All target exons are separated by gray dotted vertical lines. Selected target exons are marked. Exemplary, two randomly chosen amplicon dropouts are marked with a red arrow. **c** To illustrate the advantages of the targeted capture-based protocol over the multiplex PCR-based approach for analysis of low-quality DNA the normalized coverages (y-axis) of targets of capture-based and multiplex PCR-based NGS of two FFPE samples (P 5 and P10) are displayed at single-base resolution along all concatenated *BRCA1/2* targets (x-axis). Exemplary, two randomly chosen amplicon dropouts are marked with a red arrow

Sequence artifacts were detected with a read frequency of over 5% in all control samples with the multiplex PCR-based approach, but not with the modified capture-based approach (Additional file 2: Table S1 and Additional file 3: Table S2). We further compared the normalized nucleotide coverage in the five blood control samples (Fig. 1). The coverage at each nucleotide position was normalized against the median coverage of *BRCA1/2* targets of the individual sample. Normalized coverage was distributed more evenly among targets with the targeted capture-based strategy than with the multiplex PCR-based NGS approach (Fig. 1a). With targeted capture-based NGS normalized coverage varied less both among targets of each sample among the five blood samples.

For 13 FFPE tumor samples (Additional file 2: Table S1, P01-P13) we again found that with the modified targeted capture-based NGS all nucleotides were sufficiently covered (> 30). In contrast, application of multiplex-PCR-based NGS to the same samples resulted in low-covered (< 500) and non-covered nucleotides. Additional sequence artifacts with a frequency of over 5% of reads occurred in several samples with the multiplex PCR-based approach samples. Notably, as for blood-derived DNA, no sequence artifacts were detected using DNA derived from FFPE tissues with the targeted capture-based NGS approach.

In order to compare the enrichment-capacities of the modified targeted capture-based NGS and the multiplex PCR-based NGS for highly fragmented DNA, we applied both methods to two samples with highly fragmented DNA from FFPE samples (Additional file 2: Table S1; samples P14 and P15). The high degree of fragmentation was determined using qPCR (delta Ct values > 4, Additional file 2: Table S1). With both approaches, numerous nucleotides were not or insufficiently covered, and many sequence artifacts occurred (Additional file 1: Figure S1). The performance did not improve with repetition of both approaches, which was applied to one of the samples (Additional file 2: Table S1; sample P14). However, the targeted capture-based protocol produced less artifacts and less low- or non-covered nucleotides compared to the multiplex PCR-based method.

The normalized coverage obtained by multiplex-PCR based NGS varied greater if DNA was derived from FFPE-tissue than from blood (Fig. 1B). In contrast, the targeted capture-based approach produced even coverage of all targets of *BRCA1/2* genes irrespective of the material from which the DNA was obtained. For the purpose of illustration two exemplary data sets of two patients (P05 and P10) are depicted in Fig. 1C, showing even coverage distribution and no target drop outs with the targeted capture-based protocol applied to their low-quality DNA extracted from FFPE tissues. However, the normalized coverages strongly vary with both approaches when applied to very low quality and highly fragmented DNA, as can be seen in two cases (samples of patient P14 and P15 compared to the 13 FFPE samples, Additional file 1: Figure S1).

### The modified targeted capture-based NGS protocol improved accuracy of variant detection using DNA extracted from FFPE samples

We further analyzed whether both NGS strategies can reliably identify SNVs and InDels using FFPE ovarian cancer tissue from a cohort of 13 HBOC patients. Pathogenic germline variants in *BRCA1/2* had previously been detected in 12 of the patients by sequencing of blood-derived DNA within a diagnostic setting (Tab. 1). The 13 FFPE samples were investigated regarding pathogenic missense variants, truncating- and frameshift variants and variants with a potential splice site effect as well as variants of unknown significance (VUS), polymorphisms, and artifacts.

With the targeted capture-based NGS protocol all twelve known pathogenic mutations could be detected (Tab. 1). In contrast, one of the known variants was missed with the multiplex PCR-based protocol due to insufficient coverage (Tab. 1, P10 and Additional file 3: Table S2, P10). The multiplex PCR-based panel contains two primer sets (A and B) for two independent multiplex PCRs. Both sets of amplicons (A and B) are sequenced to identify artifacts based on their presence in only one dataset and absence in the second dataset. Therefore, in one case (patient 5), the dropout of one amplicon in one of the amplicon datasets resulted in dismissal of the pathogenic variant as an artifact (Tab. 1, P05 and Additional file 3: Table S2, P05). A very high number of insufficiently and non-covered nucleotides occurred with the multiplex PCR-based approach applied to the FFPE samples of patient P05 and P10, demonstrating low performance of the protocol in both cases (Additional file 2: Table S1, P05 and P10).

In addition, we found three low frequent (5–6% of reads) false-positive pathogenic variants (*BRCA1*:p.Ls654fs, *BRCA2*:p.Thr3033fs, *BRCA2*:p.Ile605fs) in several samples with the multiplex PCR-based approach (Additional file 3: Table S2). These variants occurred in both amplicon datasets A and B with equal frequencies. Manual inspection of the three variants revealed that they occur at poly(A) and poly(T) homopolymer stretches (*N* = 8). These variants are most likely artifacts as a result of PCR amplification errors altering mononucleotide repeat lengths of the homopolymers [27]. These artifacts did not occur with the targeted capture-based NGS approach (Additional file 3: Table S2).

All twelve variants in *BRCA1/2* were heterozygous germline mutations. In contrast, the allele frequencies of these variants in FFPE samples ranged from 69 to 83%. This is in line with the hypothesis of LOH (loss of the corresponding *BRCA1/2* wild type allele) having occurred in all tumors that were analyzed, especially considering a tumor content of about 70–80% was determined.

**Fig. 2:**
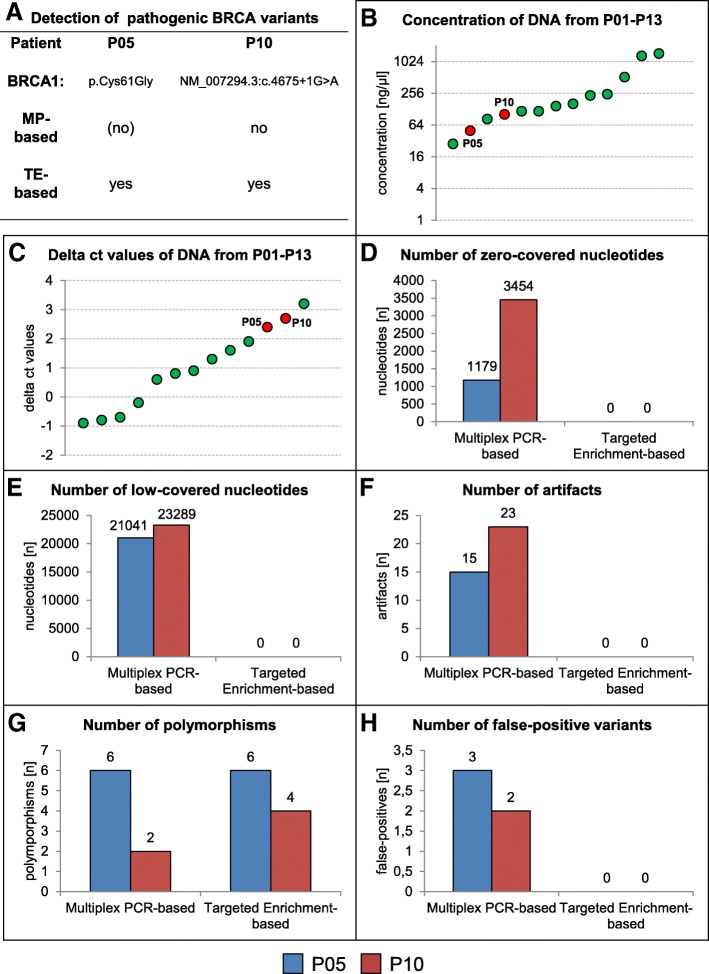
Advantages of the modified targeted capture-based protocol over traditionally applied multiplex PCR-based NGS of two low quality DNA-samples extracted from FFPE tissues of patients P5 and P10. **a** In contrast to the modified targeted capture (TP)-based approach, pathogenic *BRCA1* variants were missed by the multiplex PCR (MP)-based approach. **b** DNA concentration of FFPE samples of patients P1 to P13. The FFPE samples of P5 and P10 are marked by a red dot. **c** Delta ct values of DNA from FFPE samples of patients P1 to P13. The DNA of FFPE samples of P5 and P10 are marked by a red dot.**d** Zero-covered nucleotides occur only with the multiplex PCR-based protocol. **e** Low-covered nucleotides (< 500 with the multiplex PCR-based approach and < 30 with the targeted capture-based approach) occur only with the multiplex PCR-based protocol. **f** Artifacts only occur with the multiplex PCR-based approach. **g** Polymorphisms were detected via both approaches. In patient 10 two additional polymorphisms were found with the targeted capture-based protocol. **h** False-positive pathogenic *BRCA* variants were detected in both patients using the multiplex PCR-based technique

To summarize the advantages of our novel, capture-based protocol, Fig. 2 exemplary demonstrates the superiority of this approach over the multiplex PCR-based protocol for analysis of low-quality DNA originating from FFPE tissue of patients P05 and P10. Most importantly, we were able to identify the underlying pathogenic *BRCA1* variants in both specimens which, with the traditional protocol, was not reliably possible (Fig. 2a). Both DNAs used in this experiment are of relatively low concentration (Fig. 2b) and low quality (Fig. 2c), causing numerous low- or non-covered nucleotides in multiplex PCR-based sequencing. This was not observed with the modified targeted capture-based protocol (Fig. 2d, e). In addition, artifacts where observed in both patient datasets with the traditional multiplex-PCR based approach, while the novel capture-based protocol did not produce such artifacts (Fig. 2f). The number of polymorphisms detected by the two approaches is similar (Fig. 2g), in P10, however, two more polymorphisms were detected by the novel technique. Notably, in both patients false-positive pathogenic variants were detected with the multiplex-PCR based NGS, while no such variants were occurred with the targeted capture-based protocol (Fig. 2h).

### The modified targeted capture-based NGS strategy enabled CNV detection in *BRCA1/2* genes with high sensitivity and specificity

A major challenge is the detection of CNVs in targeted panel sequencing approaches, since only a small number of genes are being sequenced. We have shown that targeted capture-based NGS of blood- and FFPE-derived DNA generates equal sequencing quality in terms of normalized coverage distribution among *BRCA1* and *BRCA*2 targets (Fig. 1). In addition, non- or insufficiently covered nucleotides were absent (Additional file 2: Table S1). To test if reliable detection of heterozygous deletions and duplications in *BRCA1* and *BRCA2* is possible based on panel data obtained by capture-based NGS, we analyzed samples with known exon spanning deletions/duplications as well as control samples.

We first analyzed blood samples of five healthy control individuals and nine HBOC-patients, who did not appear to carry CNVs, as determined by customized high-resolution 8 × 60 k array CGH [3, 8]. In all fourteen cases no CNVs were detected via panelcn.MOPS (Tab. 2, K01-K05 and P01-P09). We then applied the pipeline to seven patients known to carry heterozygous germline *BRCA1* deletions and one patient with a *BRCA*2 duplication. CNVs had previously been detected by array CGH [8]. Application of panelcn.MOPS to NGS data of these seven test cases detected five of the six known deletions in *BRCA1* genes and the duplication in *BRCA*2 (Tab. 2, P16 to P22).

**Table 2:** Comparison of CNV detection by array CGH to CNV detection by panelcn. MOPS using blood-derived DNA

				Acurracy for calling of	
ID	Sample group	Material	array CGH^a^	CNVs^b^	no CNVs^b^
K01	Control	Blood	without pathological findings	–	0.96
K02				–	0.97
K03				–	0.97
K04				–	0.90
K05				–	0.97
P01	Control routine diagnostic	Blood	without pathological findings	–	1.00
P02				–	1.00
P03				–	1.00
P04				–	0.98
P05				–	1.00
P06				–	1.00
P07				–	1.00
P08				–	1.00
P09				–	1.00
P16	Known CNVs	Blood	BRCA1: 41.200.842–41.201.265 × 1 (loss E22)	1.00	1.00
P17			BRCA1: 41.167.511–41.338.305 × 1 (BRCA1 loss)	0.86	0.89
P18			BRCA1: 41.215.214–41.242.384 × 1 (loss E12-E18)	1.00	0.99
P19			BRCA1: 41.215.214–41.242.384 × 1 (loss E12-E18)	0.75	0.75
P20			BRCA1: 41.261.356–41.261.915 × 1 (loss Intron3)	*not possible* ^c^	1.00
P21			BRCA2: 32.891.687–32.916.514 × 3 (dup. E4–13, E27)	0.39^d^	0.97
P22			BRCA1: 41.227.803–41.258.803 × 1 (loss E4–13)	0.95	1.00

^a^ Hackman et al. *2016*

^b^For BRCA1/2 target specific panelcn.MOPS results inspect Additional file 5: Excel spreadsheet document 1

^c^CNVs restricted to introns only are not targeted via targeted capture-based NGS and panelcn.MOPS

^d^Duplication calling is more difficult compared to deletion calling and individual adjustment of log2 values might be necessary

**Fig. 3:**
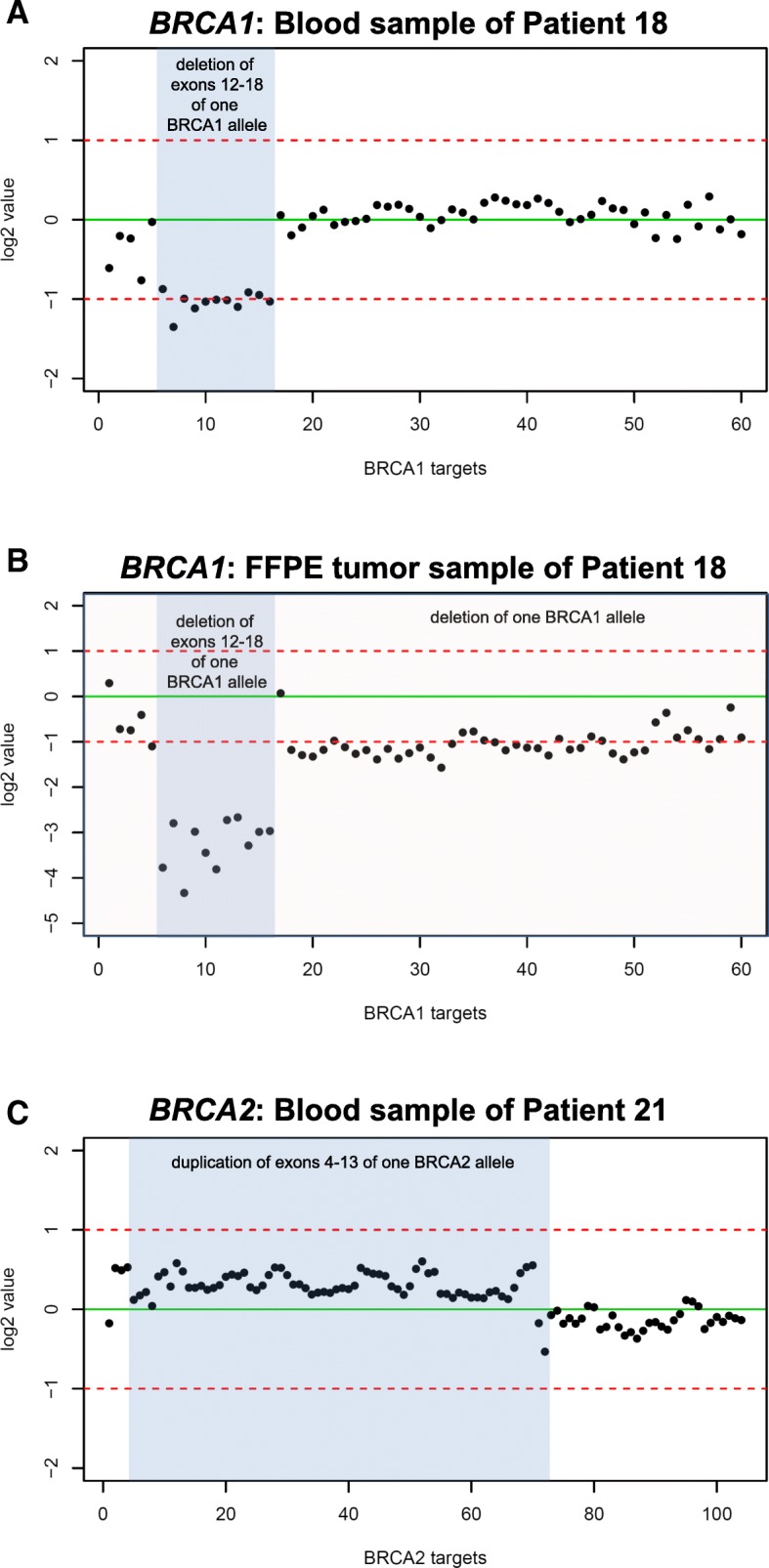
Example of NGS-based CNV detection in *BRCA1* and *BRCA2.* Illustration of CNV detection by panelcn.MOPS as performed for patient 21 using a blood (**a**) and a FFPE tumor sample (**b**). Regions (x-axis) are labelled with the individual *BRCA*-specific targets. The log2 values of the normalized read counts (RCs) of each sample are symbolized by black dots. InDels are highlighted. **a** Heterozygous deletion of exons 12 to 18 in *BRCA1* in the blood sample (pathogenic germline variant). **b** LOH of a complete *BRCA1* allele in the tumor of the same patient. The allele with detected loss of exons 12–18 remains present in the tumor. This is represented by the lower log2 ratios of corresponding *BRCA1* targets. **c** The duplication of *BCRA2* exons E4-E13 of one *BCRA2* allele in the blood of patient 24. No tumor tissue was available

The deletion of intron three in patient 20 could not be detected using the targeted capture-based NGS protocol, since intronic regions are not covered. A *BRCA1/2* target-specific listing for each sample is provided in Additional file 5: Excel spreadsheet document 1. As an example, Fig. 3a shows the heterozygous loss of *BRCA1* exons 12 to 18 in the blood sample of patient P18 detected by targeted capture-based NGS. As a second example the heterozygous germline duplication of *BCRA2* exons 4–13 carried by patient P21 is displayed in Fig. 3c.

We calculated the accuracy of CNV calling based on targeted capture-based NGS data obtained from blood samples using panelcn.MOPS. For this, the array CGH data generated from blood samples was used as a reference dataset (Tab. 2). Overall, in six of seven cases (Tab. 2, P16-P19 and P21-P22) we correctly called the CNVs previously identified by array CGH. The deletion of intron three in sample P20 (Tab. 2) was detected by array CGH but could not be seen with the NGS-based approach, since only exonic regions were targeted by the protocol (86% sensitivity for whole gene calling, 100% sensitivity for exonic calling). Analysis of the remaining 14 samples (Tab. 2, K01-K05 and P01-P09) correctly did not identify any CNVs (100% specificity). In order to determine the average accuracy, the six cases with exon-spanning CNVs were used (Tab. 2, P16–19 and P21–22). Calculation of overall accuracy resulted in 83% correct target-specific CNV calling by the NGS-based approach compared to array CGH results. The average accuracy for correct negative target-specific CNV calling was 97% (Tab. 2).

Tumor tissue from FFPE samples was available from all five patients with known heterozygous exon-spanning germline deletions in *BRCA1* (Additional file 4: Table S3, P16-P20). All five germline *BRCA1*-deletions were also detected in FFPE-derived tumor DNA by the targeted capture-based NGS combined with the CNV calling pipeline. Notably, log2-ratios for these deletions implied homozygous deletions in tumor tissue. Additionally, this approach detected heterozygous deletions of the entire *BRCA1* gene as the second hit in four of the tumor samples. For example, the tumor of patient 18 harbored only one *BRCA1* allele, which carried the loss of exon 12 to 18 that was identified in a heterozygous state in blood (Fig. 3b).

Furthermore, we applied the *BRCA1/2* CNV calling-pipeline to the data generated by NGS analysis of twelve FFPE tumor samples with known germline variants (Table 1, Additional file 4: Table S3, P01–13). We thereby identified deletions of *BRCA1* or/and *BRCA2* in all cases, proving that the observed LOH in these cases was caused by deletion of the wild type *BRCA1/2* alleles (Additional file 4: Table S3).

### Genetic testing using FFPE tissue from deceased index patients to aid clinical decision making in HBOC families

We applied the adjusted targeted capture-based strategy for *BRCA1/2* variant detection, including CNV detection, to DNA extracted from FFPE-tissues of 11 deceased index patients from 11 HBOC families. The underlying genetic alterations underlying tumor predisposition in these families was unknown (Additional file 4: Table S3, P23-P33). In one of the samples, surrounding normal tissue was micro-dissected from the tumor block and was used for analysis (Additional file 4: Table S3; P27), while in the other cases only tumor tissue was available.

Pathogenic variants in *BRCA1/2* were identified in four of these 11 samples, including the patient from whom non-tumor tissue was analyzed (P27). The three tumor samples (P26, P28, P29) had homozygous pathogenic variants in either *BRCA1* or *BRCA2* based on variant frequencies, indicating LOH in these samples. We confirmed deletion of the wild-type allele in all three cases by applying the targeted-capture base CNV detection approach. A pathogenic *BRCA2* mutation was found in heterozygous state and therefore interpreted as likely germline mutation.

**Fig. 4:**
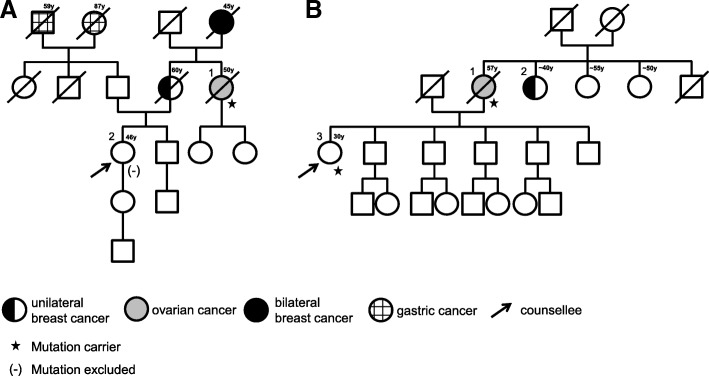
Pedigree of two families fulfilling the criteria for hereditary breast and ovarian cancer. **a** In this family, all patients that had developed cancer were deceased. We identified a pathogenic *BRCA2*-mutation NM_000059.3:c.7879A > T,p.(Ile2627Phe) in paraffin-embedded normal tissue from individual 1 (P27). The counselee (2) did not inherit the mutation and could therefore be relieved from her concern to have inherited the genetic predisposition from her mother. **b** In this family, patient 2, suffering from breast cancer, refused genetic testing. The pathogenic *BRCA2*-mutation NM_000059.3:c.8167G>C, p.(Asp2723His) was identified in tumor tissue available from individual 1 (P28). Targeted analysis revealed that the daughter (3) also carried the mutation, confirming that the variant identified in tumor tissue from the mother was indeed a germline variant. The daughter was therefore included in a high risk breast- and ovarian cancer screening program

We offered predictive testing to all relatives for the *BRCA1/2* mutations identified in the four index patients. Overall, 12 healthy individuals and two individuals affected by breast cancer were available for testing and the mutation was confirmed in eight of them. In all three families in which a mutation was identified by analysis of tumor tissue of the deceased index patient (P26, P28, P29), at least one family member also carried the pathogenic mutation, thus confirming that these were indeed germline mutations. All female mutation carriers were offered to participate in a clinical surveillance program for woman at high risk for breast and ovarian cancer. Six individuals did not carry the familial mutation and could therefore be excluded from high-risk specialist screening programs. The pedigrees of two of the families are exemplary depicted in Fig. 4 (A: P27 and B: P28). These two pedigrees represent the two clinical scenarios that were encountered: A pathogenic germline mutation was identified in normal tissue from the previously deceased mother. The daughter, who had been undergoing annual intensive breast cancer surveillance based on her calculated high lifetime risk, did not inherit the mutation and could therefore be relieved from this burden. In the family of P28, the healthy daughter of the index patient also carried the mutation that was identified in her mothers´ tumor and was therefore included in a high-risk breast- and ovarian cancer screening program.

In addition to the four high frequent variants identified in the four index patients P26-P29, low frequent pathogenic variants (5–13% of mapped reads) that passed our control criteria (frequency ≥ 5%, coverage ≥30) were detected in two samples without high-frequent *BRCA1/2* mutations (P24, P25) and additionally in sample P28 (which also carried the missense mutation *BRCA2*:p.Ile2627Phe with a frequency of 98%) (Additional file 4: Table S3). One of these low-frequency variants is classified as potential artifact caused by a poly(A) homopolymer stretch (*BRCA2*:p.Asp1781fs in P25), while the other low frequent variants were regarded as possibly true variants after manual evaluation of the read mappings. With regards to the low frequency of these mutations it seems reasonable to assume they might have occurred somatically in tumor subclones.

Of the seven tumor samples without high frequent *BRCA1/2* SNVs (Additional file 4: Table S3, P23-P25 and P30-P33) all but one case (P31) had heterozygous deletions of the complete *BRCA1* and/or *BRCA2* gene detected by CNV-calling. This is notable since of the ten tumors that were sequenced, the only tumor without a deletion of *BRCA1/2* (P31) was also the only benign one (a lipoma), while the other ten tumors were malignant ovarian-, breast- and pancreatic tumors. One can speculate that the deletions of *BRCA1* and/or *BRCA2* observed in these samples might be somatic alterations, however, we could not validate this, since no healthy tissue or blood was available for germline testing.

Moreover, we sequenced the TruSight Cancer panel (Illumina, Inc.), which in addition to *BRCA1/2* targets another 92 genes. By this, pathogenic mutations in other cancer related genes were identified in three of the samples without BRCA SNVs: a pathogenic germline mutation in *PALB2* NM_024675.​​3:​​c.​​509_510delGA,​​ p.​​(Arg170Ilefs*14) in P30, in *TP53* NM_000546.5:​c.​​587G>C, p.(Arg196Pro) in P31, and in *NBN* NM_002485:​c.​657_661delACAAA, ​p.​(Lys219Asnfs*16) in P33, respectively. In all three cases, germline status of the mutation could be confirmed by testing of normal, non-tumorigenic tissue. Consequently, predictive testing was offered to the family members. In P33 testing of the parents revealed that the *TP53* mutation must have occurred de novo in in this patient. Two relatives of P31 could be tested so far, but both did not inherit the mutation. Predictive testing in the family of P30 confirmed that two daughters inherited the *PALB2* mutation from their father. Both were included in specialist screening programs and have now been diagnosed with breast cancer.

## Discussion

In this study we demonstrate that an adjusted targeted capture-based NGS strategy performs superior to the routinely applied multiplex PCR-based NGS protocol for genetic variant detection (including CNVs) in *BRCA1/2* in blood and, in particular, in FFPE-tissues. Dropout of amplicons, insufficiently covered nucleotides, false-positives pathogenic variants and further sequence artifacts were primarily seen with the multiplex PCR-based approach, impeding the detection of pathogenic *BRCA1/2* mutations in two cases.

False positive pathogenic variants may include deamination of cytosine bases in DNA extracted from FFPE samples, apurinic/apyrimidinic sites, PCR amplification introduced errors and sequencing errors [27, 28]. Manual exploration of multiplex PCR-based sequence data revealed that false-positives pathogenic variants mostly represented PCR amplification introduced errors at poly(A) and poly(T) stretches [27]. This emphasizes the importance of careful evaluation of the read mappings in order to distinguish true mutations from artifacts, which might otherwise misguide clinical decision making. Since the false-positive pathogenic variants detected by multiplex PCR-based NGS occurred at defined poly(A) and poly(T) homopolymer regions in *BRCA1/2* they can be classified as artifacts and consequently can be filtered out bioinformatically in subsequent analyses. This is of special importance for the analysis of DDR-genes such as *BRCA1/2,* since cancer-predisposing loss-of-function mutations may occur at any position in the gene. In contrast, in oncogenes only a restricted number of hot spots are of diagnostic interest. Moreover, amplicon dropouts occurring at positions with pathogenic variants d(as demonstrated for two samples in this study) can cause false negative results. The insufficient coverage of single nucleotides observed with multiplex PCR-based NGS may impede reliable variant detection. Insufficiently covered nucleotides did not occur with the targeted capture-based NGS strategy. Recently, Illumina Inc. offered the TruSight Tumor 170 panel targeting 170 genes including *BRCA1 and BRCA2*. The panel also relies on a targeted capture-based protocol that is applicable on DNA originating from FFPE tissue and might therefore be suitable for our protocol modification steps.

Bioinformatics pipelines are being developed in order to increase accuracy of multiplex PCR-based NGS data and subsequent variant calling [29]. However, targeted capture-based protocols represent an alternative for genetic analysis of FFPE tissues. In a recently published study, HaloPlex targeted enrichment and Illumina NGS technology were used in order to determine germline variants in *BRCA1* and *BRCA2* genes in FFPE samples of non-tumorigenic tissue [9]. It was estimated that germline *BRCA1/2* variant detection is possible in archived FFPE tissue samples after up to 30 years [9]. FFPE quality, different DNA extraction protocols and level of DNA integrity testing all individually influence the quality of extracted DNA. It is therefore recommended that each lab should evaluate different DNA extraction and amplification protocols independently and choose the method most suitable for the lab’s workflow using reference material [30]. In addition, individual artifacts caused by the selected method have to be known in each lab to avoid reporting of false-positive results [31]. Using the targeted capture-based approach, the complete coding sequences of the large *BRCA1* and *BRCA2* genes can be analyzed and variant detection does not have to be restricted to selected regions of the gene.

A fourth advantage of our novel method is that CNV detection can be performed for *BRCA1/2* solely based on NGS panel data from FFPE materials. At the Institute for Clinical Genetics at TU Dresden, a custom array CGH is used to detect pathogenic germline CNVs in hereditary cancer susceptibility genes in blood samples [8]. However, carrying out additional array CGH to obtain copy number information not only causes extra costs, but its applicability is limited to high-quality DNA. CNV detection based on FFPE-derived DNA has previously been performed using the Affymetrix Cytoscan HD Chip for eight lymphoma samples [32]. In another setting 13 FFPE tissue samples were analyzed for CNVs using whole genome, whole exome, and targeted exon sequencing [25]. We show that the application of capture-based panel sequencing combined with panelcn.MOPS [21] is suitable for the detection of deletions and duplications in *BRCA1/2* with high resolution up to single exon level in both blood and FFPE tissues. Sensitivity and specificity for detection of coding region variants were determined at 100%. Therefore, in our opinion, the targeted capture-based NGS complemented with panelcn.MOPS offers a strategy for preliminary CNV-testing, covering copy number changes of single exons up to all targeted exonic regions in *BRCA1/2* genes. The approach works well for analysis of blood-derived DNA and, if necessary, also FFPE tissue-derived DNA. We suggest subsequent validation of positive results by array CGH, if possible, until the application of panelcn.MOPS or a similar tool has been proven to be as reliable as array CGH. One limitation of our NGS-based protocol is that CNVs occurring in intronic regions cannot be detected, since only exonic regions are targeted by the NGS panel used in this approach. This limitation could be overcome by the use of probes targeting also the intronic regions of targeted genes.

The multiplex PCR-based strategy is not well suited for CNV calling as it causes amplicon dropouts, insufficiently covered nucleotides and in inhomogeneous normalized coverage of target regions. In addition, the R package panelcn.MOPS currently does not support the application to NGS data generated by multiplex PCR-based panel sequencing [21]. We tested another R package (CNVPanelizer) that is suitable to detect CNVs in multiplex PCR-based approaches [33]. However, using standard parameters, only CNVs affecting the whole gene can be detected. We subdivided *BRCA1/2* with respect to their target regions to increase resolution of CNV detection by CNVPanelizer but did not observe any improvement (data not shown). Although detection of highly amplified or lost coding regions in tumors can be detected based on multiplex PCR-based NGS data [34], no reliable tools are available to detect small deletions or duplications with this type of data. Based on our observation of inhomogeneous coverage and allele-drop outs it seems very challenging to perform CNV calling in a routine diagnostic setting based on multiplex PCR-based panels with only a small number of targeted genes. Alternative NGS data-based bioinformatics approaches such as Hidden Markov Models (HMMs) with three states (deletion, unchanged, amplification) [35, 36] might be a valid alternative in the future, after this approach has further been optimized and validated.

One limitation of the target capture protocol is that a larger amount of DNA is necessary (at least 50–100 ng) compared to multiplex PCR-based approaches requiring less DNA input (at least 10 ng). Moreover, less hands-on-time is necessary for the multiplex PCR-based approach than for the targeted capture-based protocol.

An important advantage of the target-capture strategy compared to the multiplex-based approach is that a larger target region can be covered. For example, the TruSight Cancer panel includes altogether 94 cancer related genes. Therefore, the analysis of pathogenic variants, including CNVs, is possible not only in *BRCA1/2*, but also in an additional 92 further cancer-related genes. This allows for genetic testing for pathogenic germline variants to a much broader extend. For this reason we were able to identify two additional tumor cases with mutations in other DNA damage response genes than *BRCA1/2* (*PALB2* and *NBN*), which might also respond to PARP-inhibitor therapy. *BRCA1/2-*mutated ovarian cancers respond to platinum-based chemotherapy and PARP-inhibition therapy and current studies further investigate the effect of PARP-inhibition both on other cancer entities and on cancers driven by pathogenic mutations in other DDR-genes [37–39]. With regards to this, detection of SNVs and CNVs in a larger number of genes becomes increasingly important.

We furthermore demonstrate the clinical applicability of our approach to identify pathogenic germline mutations in FFPE tissue from deceased index patients. We were thereby able to identify pathogenic germline *BCRA1/2* mutations in four of eleven HBOC families in which the underlying mutation was previously unknown and could therefore confirm that these cases indeed were the result of an inherited syndrome. This has important consequences for the families, since only if the underlying mutation is known can predictive testing be offered to healthy family members. In families suspicious for HBOC but with no causative mutation identified, healthy relatives are included in or excluded from clinical surveillance programs solely based on calculations of mutation carrier risks [40, 41]. By this strategy many women who do not actually carry a mutation receive yearly gynecologic examinations and body-imaging, condoning both the psychological burden and the radiation exposure that comes with these measures, while other women, further away from the index patient in the pedigree, might not get clinical surveillance although they do carry the mutation. For such families, where the index patients who suffered from cancer are deceased, analysis of stored FFPE tissue might be the only option to identify the predisposing genetic lesions.

## Conclusion

We show that an adjusted targeted capture-based NGS protocol can reliably identify SNVs, small InDels and CNVs with high resolution up to single-exon level using DNA from FFPE tissues. With regards to the growing demand for FFPE tissue analysis, especially for the detection of *BRCA1/2* mutations to guide clinical decision making and to identify patients with hereditary cancer syndromes, we consider optimization of CNV detection a major step in ensuring sufficient patient care. Moreover, our capture-based approach allows sequencing of a larger region of interest than multiplex PCR-based protocols, which not only makes possible homogenous normalized coverage as a requirement for reliable CNV-calling but also enables analysis of further DNA-damage response genes such as *PALB2,* which has important implications for patient management*.*

## Additional files

Additional file 1: **Figure S1.** Comparison of normalized coverage of targeted capture-based NGS to multiplex PCR-based NGS applied to FFPE tumor samples. **A:** Normalized coverage (y-axis) of targeted capture-based and multiplex PCR-based NGS of FFPE samples from patient 1 to 13 (Additional file 3: Table S2, P01 to P13) at single-base resolution along all concatenated *BRCA1/2* targets (x-axis). Samples P01 to P13 are color-coded in blue. **B:** Normalized coverage (y-axis) of targeted capture-based and multiplex PCR-based NGS of FFPE samples from patient P14-P15 (Additional file 3: Table S2, P14 to P15) of FFPE samples from patient P14-P15 (Additional file 3: Table S2, P14 to P15) of FFPE samples from patient P14-P15 (Additional file 3: Table S2, P14 to P15) of FFPE samples from patient P14-P15 (Additional file 3: Table S2, P14 to P15) at single-base resolution along all concatenated *BRCA1/2* targets (x-axis). Samples are color-coded in red and represent highly fragmented DNA. Exemplary, randomly chosen capture target dropouts and amplicon dropouts are marked by a red arrow. The vertical green line indicates the end of *BRCA2* targets (target number is increasing from left to right which corresponds to five to three prime orientation of the gene) and the start of *BRCA1* targets (target number is decreasing from left to right which corresponds to five to three prime orientation of the gene). The horizontal green line displays normalized coverage of 1.0. All target exons are separated by gray dotted vertical lines. Selected exons are marked. (PDF 2100 kb)

Additional file 2: **Table S1** Summary of control (K01 to K05) and patient (P01 to P33) samples used for comparison of the performance of targeted capture-based NGS to multiplex PCR-based NGS. (XLSX 11 kb)

Additional file 3: **Table S2** Pathogenic *BRCA1/2* variants in investigated blood and FFPE samples. (XLSX 12 kb)

Additional file 4: **Table S3** Pathogenic variants and CNVs in FFPE samples from all patients (P01 to P33). (XLSX 18 kb)

Additional file 5: Excel Spreadsheet 1 Sample-specific panelcn.MOPS output at *BRCA*1 and *BRCA*2 target regions generated by the targeted capture-based NGS approach applied to DNA originating from blood and FFPE samples from all patients (P01 to P33) compared to array CGH results (if available). (XLSX 1143 kb)

## Abbreviations

ADP: Adenosine Diphosphate
BRCA1: Breast Cancer Gene 1
BRCA2: Breast Cancer Gene 2
CGH: Comparative Genomic Hybridization
CNV: Copy Number Variation
DDR: DNA Damage Response
DNA: Deoxyribonucleic Acid
FFPE: Formalin-Fixed Paraffin-Embedded
GenDG: German Gene Diagnostics Act
GRCh37: Genome Reference Consortium Human Build 37
HBOC: Hereditary Breast and Ovarian Cancer
hg19: Human Assembly GRCh37
HMM: Hidden Markov Models
InDels: Insertions-Deletions
LOH: Loss-of-Heterozygosity
MLPA: Multiplex Ligation-Dependent Probe Amplification
NBN: Nibrin
NGS: Next Generation Sequencing
PALB2: Partner and Localizer of BRCA
PARP: Poly(ADP-Ribose)-Polymerase 1
PCR: Polymerase Chain Reaction
SNV: Single Nucleotide Variation
TNM: TNM Classification of Malignant Tumors
VUS: Variance of Unknown Significance
