# Supplementary material for: Targeted capture-based NGS is superior to multiplex PCR-based NGS for hereditary BRCA1 and BRCA2 gene analysis in FFPE tumor samples
Source: BMC Cancer. 2019 Apr 27;19:396. doi: 10.1186/s12885-019-5584-6 (PMC6487025; doi:10.1186/s12885-019-5584-6)
Supplement: Supplementary file 1 — Figure S1. Comparison of normalized coverage of targeted capture-based NGS to multiplex PCR-based NGS applied to FFPE tumor samples. A: Normalized coverage (y-axis) of targeted capture-based and multiplex PCR-based NGS of FFPE samples from patient 1 to 13 (Additional file 3: Table S2, P01 to P13) at single-base resolution along all concatenated BRCA1/2 targets (x-axis). Samples P01 to P13 are color-coded in blue. B: Normalized coverage (y-axis) of targeted capture-based and multiplex PCR-based NGS of FFPE samples from patient P14-P15 (Additional file 3: Table S2, P14 to P15) of FFPE samples from patient P14-P15 (Additional file 3: Table S2, P14 to P15) of FFPE samples from patient P14-P15 (Additional file 3: Table S2, P14 to P15) of FFPE samples from patient P14-P15 (Additional file 3: Table S2, P14 to P15) at single-base resolution along all concatenated BRCA1/2 targets (x-axis). Samples are color-coded in red and represent highly fragmented DNA. Exemplary, randomly chosen capture target dropouts and amplicon dropouts are marked by a red arrow. The vertical green line indicates the end of BRCA2 targets (target number is increasing from left to right which corresponds to five to three prime orientation of the gene) and the start of BRCA1 targets (target number is decreasing from left to right which corresponds to five to three prime orientation of the gene). The horizontal green line displays normalized coverage of 1.0. All target exons are separated by gray dotted vertical lines. Selected exons are marked. (PDF 2100 kb) [file 12885_2019_5584_MOESM1_ESM.pdf]

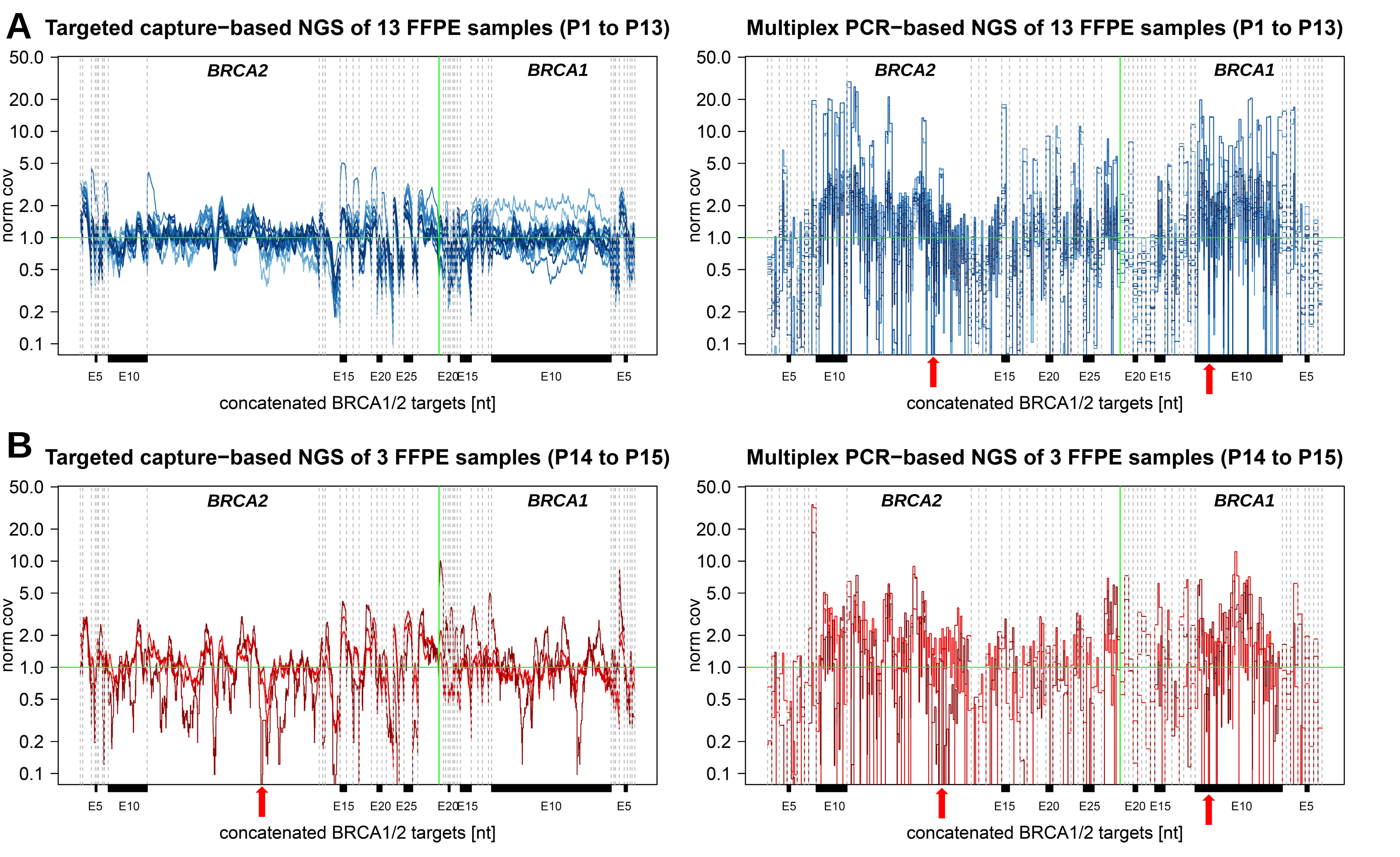
A
Targeted capture−based NGS of 13 FFPE samples (P1 to P13)
Multiplex PCR−based NGS of 13 FFPE samples (P1 to P13)
B
Targeted capture−based NGS of 3 FFPE samples (P14 to P15)
Multiplex PCR−based NGS of 3 FFPE samples (P14 to P15)
BRCA2
BRCA1
norm cov
50.0
20.0
10.0
5.0
2.0
1.0
0.5
0.2
0.1
E5
E10
E15
E20
E25
concatenated BRCA1/2 targets [nt]
